# Cost-utility analysis of a national project to reduce hypertension in Israel

**DOI:** 10.1186/1478-7547-5-16

**Published:** 2007-11-28

**Authors:** Chaim Yosefy, Gary Ginsberg, Reuven Viskoper, Dror Dicker, Dov Gavish

**Affiliations:** 1 The Israeli Forum for Prevention of Cardiovascular Disease, Barzilai Medical Center Campus Ashkelon, Ben-Gurion University, Israel; 2 Department of Medical Technology Assessment, Ministry of Health, Israel; 3 Department of Internal Medicine D, Hasharon Medical Center, Petach-Tikva, Tel-Aviv University, Israel; 4 Department of Internal Medicine A, Wolfson Medical Center, Holon, Tel-Aviv University, Israel

## Abstract

**Background:**

This study aims to calculate the health effects and costs of a proposed national hypertension prevention and control program.

**Methods:**

Interventions are based on experience from our two programs: 10-year period of Ashkelon Hypertension Detection and Control Program (AHDC Program) and the Israel Blood Pressure Control (IBPC) program. The costs of a nationwide program were calculated based on economic data, training staff levels, course frequency and unit costs.

**Results:**

Over the next 20 years, the program should decrease the risk in one-half of the treated hypertensive cases of the following ailments: cardiovascular events such as Acute Myocardial Infarction (AMI) and Unstable Angina Pectoris (UAP) by 16.0%, stroke by 41.2%, End stage renal disease (ESRD) by 50.0% and peripheral vascular disease (PVD) by 42.6%. In total, around 2,242 lives, 35,117 years of life or 24,433 disability adjusted life years will be saved due to decreased mortality.

Program costs amount to $352.7 million. However savings ($537.6 million), from reduced medical treatment ($444.3 million) and reduced pharmaceutical use ($93.3 million) as a result of morbidity decreases, exceed costs by $185.0 million.

**Conclusion:**

The project which saves both lives and resources should be extended nation-wide to reach as wide a population as possible.

## Background

Cardiovascular (CV) risk factors such as hypertension, hyperlipidemia and diabetes, are insufficiently managed, according to current guidelines [1]. This mismanagement occurs in spite of the accepted fact that reaching the recommended target goals of the various guidelines is an effective means to reduce morbidity and mortality from atherosclerosis and vascular events [2,3]. Reasons for this shortfall have been described in many recent studies, showing that both the American [4,5] and the European [6,7] guidelines, are not truly implemented.

As in other developed nations, the burden of disease attributable to Hypertension, heart disease and stroke in Israel is considerable. These diseases are also among the most expensive diseases to treat [8]. In response to these important issues of disease burden in terms of human suffering and treatment cost, we initiated the Ashkelon Hypertension Detection and Control Program (AHDC Program): a community approach to reducing cardiovascular mortality [9]. During a 10-year period (1980–1990), we operated a professional doctor-nurse screening team, who were instructed to find those at high risk in control of their risk factors, in order to reduce CV morbidity and mortality. They examined 12,202 subjects, (mean age 51 +/- 7 years, range 20–65 years) accounting for 23.4% of the total regional population. High risk subjects underwent an intensive CV risk factor control program. Subjects (3,506 or 28.6%) were found to have one or more CV risk factors (hypertension, obesity, smoking, hypercholesterolemia). During an average of 2 years, follow-up BP, weight reduction, and smoking cessation remained statistically significant. Total cholesterol was unchanged. Over this period, the standardized mortality ratio (SMR) in the area for acute MI fell from 100 to 76 (P < 0.01), for CV disease from 129 to 107 (P < 0.0001), and for hypertension from 121 to 87 (P < 0.1 NS). The project saved many life-years at no additional net cost to society, and cost-effectiveness analysis showed positive results. Our community approach with mainly non pharmacological treatment was proven to be feasible and cost-effective in reducing CV morbidity and mortality. Subsequent we conducted the Israel Blood Pressure Control (IBPC) program [10], in the year 2000 in 30 general practice clinics in Israel. After 1 year of intervention in 4848 patients according to our method [10], there was a significant increase in control of risk factors. In hypertension this percentage raised from 29.0 to 46.7%, in control of body weight from 36.7 to 43.8%, in Low Density Lipoprotein (LDL) levels from 31.2 to 41.7% while the percentage of patients with glucose levels above 200 mg/dl dropped from 5.2 to 3.1%. Encouraged by these results we decided to propose a nationwide program that could also be extended to act in cooperation with neighboring countries. This study was initiated to help attain this goal and convince the health authorities, by presenting the costs and the utilities if the Ashkelon project were to be extended nationwide.

## Methods

Interventions will be based on experience from the following two programs:

### Ashkelon Hypertension Detection and Control Program (AHDC Program)

During a 10-year period (1980–1990 screening team, (MD and RN) acted to detect those at high risk to develop CV disease in order to reduce CV morbidity and mortality. They examined 12,202 subjects, (mean age 51 +/- 7 years, range 20–65 years) accounting for 23.4% of the total regional population. High risk subjects underwent an intensive CV risk factor control program [9].

### Israel Blood Pressure Control (IBPC) program

The IBPC program was initiated in the year 2000. in this survey, we included patients from 30 general practice clinics across Israel, directed by specialists in family medicine, each seeing 1000–5000 patients. The authors presented the program to all available general practice clinics in Israel. Those physicians who showed readiness to join the program were enrolled on the study [10].

### Costs of program

The costs of a nationwide program to reduce hypertension in Israelis aged 25–64 years old were calculated based on economic data, training staff levels, course frequency and unit costs (Appendix I). All costs are presented at April 2005 price levels using a 3% discount rate for the period 2005–2024. The staff education costs included educating two members of staff from each ambulatory clinic, operational costs (including tele-medicine) as well as two half-days a year of courses to maintain the level of staff education. Additional treatment costs of patients were calculated for the estimated 320,516 hypertensive cases [11,12] aged 25–64 living in Israel not only for the initial year, based on $230 per hypertensive cases less the costs of current existing utilization of 4 visits per year by 25% of all hypertensive cases. The costs of maintenance therapy were added, amounting to a discounted sum of $208 per treated person. The program will incur extra drug costs as 50% of hypertensive cases will be prescribed the correct preventive regimen of Ace Inhibitor, Aspirin and Satins costing around $0.92 per day. The remaining 50% would be treated by non-pharmacological methods.

### Incidence and mortality reductions

Disease treatment costs, both in the presence and absence of the intervention program were based on spreadsheet modeling combining unit treatment costs (Appendix I), epidemiological (Appendix II), treatment efficacy (Appendix II) and demographic factors (Appendix III). Similarly Disability Adjusted Life Years (DALY) lost from morbidity and mortality of diseases in the presence and absence of the program were calculated using the data contained in appendices II and III. All costs and utilities used a 3% discount rate for the period 2005–2024.

Based on previous experience [9,10,13], it was assumed that 50% of the persons participating would be successfully treated. Using a spreadsheet, the program benefits were estimated in terms of incidence and mortality reductions in hypertensive cases [2] over the period 2005–2024 (see Table 1) by gender and age group [25-44, 45-54, 55-64], as follows:

**Table 1:** Key Epidemiologic Variables. [Acute Myocardial Infarctions (AMI), Cerebrovascular Accident (CVA), End Stage Renal Disease (ESRD) and M/F = Male/Female].

**Age/Sex**	**Hypertension (%)**	**AMI (20 y %risk)**	**CVA (rate/1000)**	**ESRD (rate/100,000)**
25–44 M/F	3.8/3.2	5–22	1.14	NA
45–54 M/F	14.7/13.9	23–32	2.28	31.9/16.7
55–64 M/F	34.5/27.8	33–50	5.11	77.8/44.6
65<	50<	50<	9.86	148.7/88.5

#### AMI & UAP

Incidence data was based on national survey data [14], 20 year age-specific AMI and UAP risks [11] were conservatively assumed to increase gradually by 1% annually to reflect an increase in other risk factors (eg: obesity). A 16% decrease in relative risk of AMI and UAP was assumed to occur in those 50% who were successfully treated, based on Israeli results [9,10] that corroborated international findings [15-17]. One year post-AMI or Post-UAP case fatality rates [18] were adjusted by age and gender specific national background mortality rates to estimate the one year post-AMI excess mortality rates.

#### CVA

Incidence data and 20 year age-specific risks were based on Tel-Aviv stroke registry data [19]. The 1.7 relative risk of strokes in hypertensive cases [19] translates into a potential 41.2% reduction (1–1.0/1.7) of strokes in successfully treated hypertensive cases. As one year post-hospitalization mortality rates were not available, in-hospital mortality rates were used to estimate excess mortality [19].

#### ESRD

National age and gender specific incidence rates of dialysis patients and post renal transplants were obtained [20]. The relative risks (of 2.00) of ESRD in hypertensive cases (with blood pressure >140/>90) and persons with high normal blood pressure (with blood pressure of 130 to 139/85 to 89 mmHg.) compared to persons with blood pressure <130/85 were based on a recent study of 316,675 adults [21]. Incidence and relative risks were used to calculate 20 year age-specific risks for hypertensive cases. Under the assumption that 25% of hypertensive became normotensive cases and 25% became controlled (9,10), as defined to be below 140/90 mmHg according to the JNC VII guidelines [22], then the project will reduce ESRD by around 25.0%. One year ESRD mortality rates were used to attribute mortality due to hypertension [20].

#### PVD

Twenty year PVD risks were calculated by adjusting the age and gender specific 20 year CVA risks by the relative incidence of PVD to CVA [23]. Two groups of 25% of treated hypertensive cases were assumed to become normotensive cases and controlled hypertensive cases with a risk reduction of 56.8% and 28.4% respectively for PVD [23]. No excess mortality was attributed to PVD.

### Impact on treatment costs

Disease, age and gender specific decreases in incidence due to the program were multiplied by the average disease specific treatment costs obtained either from patient surveys [14], the Ministry of Health's official price list or from the accounts department of the Barzilai Medical Center. (Appendix I) and the standard protocols for hospital and post-hospital treatment (Appendix III).

#### AMI & UAP: AMI & UAP

National survey data was obtained on disease specific treatment interventions [14]. Costs were based on the budget backed-up assumption (of the Medical Technology Assessment Division of the Ministry of Health) that Cyphers would account for 40% of all simple stent and Cypher usage in the future.

#### CVA

Based on experience in Barzilai Medical Center, after 8 days average length of stay, 20% of stoke patients are discharged for three months average stay in a rehabilitation facility, after which they receive 3 months physiotherapy in a home setting. The remaining 80% of patients are discharged straight to three months physiotherapy in a home setting. Post hospitalization drug usage was assumed to be as for AMI.

#### ESRD

Currently 5.9% of ESRD patients have received transplants [20] and the rest are on dialysis (88% on haemodialysis, 12% on Peritoneal dialysis) for an average of 5.05 years [20]. Time on dialysis fell with age from 9.70 years (under 44 years) and 6.11 years (45–64) to 3.59 years (65+).

#### PVD

Treatment costs were based on an estimated of 3.5 outpatient visits a year over 10 years for treatment of PVD, based on current Israeli protocols and practice for monitoring PVD patients for any deterioration, every 3 to 4 months.

### Disability Adjusted Life Year (DALYs) saved

DALY gains (subject to a 3% discount rate) were calculated by aggregating the gains from decreased mortality and morbidity as a result of the program's intervention. The average health state valuation (HSV) is less than one, especially in older age groups where people have multiple dysfunctions. In order to reflect this, DALYs saved from averted deaths were calculated by multiplying the disease specific life year gains by the age and gender specific healthy adjusted life expectancies (HALE) [24].

Age-specific HSVs for disease conditions were obtained from the WHO global burden of disease study [25]. Total DALYs loss from morbidity was obtained by multiplying age-specific disease incidence by the average disability loss (1-HSV) and by the time spent in the morbid condition, obtained from the WHO [26] or from the survey of dialysis patients for ESRD [19]. For PVD disease duration was assumed to be for 10 years with a HSV of 0.975 (or disability weight of 0.025).

### Cost per DALYs ratio

The net cost of the program was calculated by subtracting the reduced treatment costs (including reduced use of pharmaceuticals) from the running cost of the program. The major cost-effectiveness indicator is the Cost per DALYs ratio calculated by dividing the net cost of the project by the expected number of DALYs saved. This was only able to be calculated where the project was not cost-saving.

Projects are considered to be cost saving if their net cost is negative, very cost-effective if the cost per DALYs is less than the gross domestic product (GDP) per capita, cost-effective if the cost per DALY is between one and three times the GDP per capita and not cost-effective if the cost per DALYs exceeds three times the per capita GDP [27].

## Results

The program is expected to result in their being 8,749 fewer AMIs, 8,134 fewer PVDs, 8,074 fewer CVAs, 6,765 fewer UAPs and 2,316 fewer cases of ESRD (Table 2). Around half of the estimated total of 2,242 fewer deaths will be due to the reduction in cerebrovascular strokes (1,114 fewer deaths), while there will be 543 fewer deaths from AMIs, 440 fewer deaths from ESRD and 145 fewer deaths from UAP (Table 2). These translate into a total of 35,117 life years saved, around 48.1% due to decreased CVA (Table 3). After adjusting for disabilities, around 18,726 discounted DALYs (24,433 undiscounted) are saved from reduced mortality, of whom 48.6% are due to reduced CVA, 24.6% from fewer AMIs, 20.6% from reduced ESRD and 6.3% from reduced UAP (Table 3).

**Table 2:** Impact of Hypertension Control Program on incidence and mortality (2005–2024). [Acute Myocardial Infarctions (AMI), Unstable Angina Pectoris (UAP), Cerebrovascular Accident (CVA), End Stage Renal Disease (ESRD) and Peripheral Vascular Disease (PVD)].

	**INCIDENCE Without Program**	**INCIDENCE With Program**	**Decrease**	**MORTALTY Without Program**	**MORTALITY With Program**	**Decrease**
AMI	98,182	89,433	8,749	6,094	5,551	543
UAP	38,939	32,174	6,765	696	552	145
CVA	39,217	31,143	8,074	5,412	4,298	1,114
ESRD	9,277	6,961	2,316	1,763	1,323	440
PVD	38,194	30,060	8,134	NA	NA	NA

TOTAL	NA	NA	NA	13,965	11,723	2,242

**Table 3:** Life years and discounted DALYs saved (2005-2024) of hypertension program. [Acute Myocardial Infarctions (AMI), Unstable Angina Pectoris (UAP), Cerebrovascular Accident (CVA), End Stage Renal Disease (ESRD) and Peripheral Vascular Disease (PVD), Disability Adjusted Life Years (DALYs)].

		**Mortality**	**Morbidity**	**Total**
	**Life Years**	**DALY**	**DALY**	**DALY**

AMI	8,925	4,611	159	4,770
UAP	2,401	1,174	330	1,504
CVA	16,904	9,091	10,812	19,903
ESRD	6,888	3,850	783	4,633
PVD	-	-	1,558	1,558

TOTAL	35,117	18,726	13,643	32,369

A smaller quantity of DALYs (13,643 discounted and 17,801 undiscounted) will be saved from reduced morbidity. While the large majority of morbidity gains (around 61.5%) are due to decreased CVA incidence, AMI/UAP and ESRD account for a further 14.3% and 14.3% respectively (Table 3).

In total, around 32,369 discounted DALYs will be saved, around 57.9% being attributable to reduced CVA mortality. Program costs amount to $352.7 million, including $9.8 million for staff education, $19.2 million for telemedicine and operational expenses, $70.0 million for patient training during the first year of treatment, $66.8 for patient maintenance visits in subsequent years and $186.2 million for additional use of pharmaceuticals.

Averted renal dialysis and transplants accounted for the majority (70.2%) of the $ 444.4 million savings in reduced medical treatment costs. In addition there were $93.3 million savings from reduced pharmaceutical use as a result of morbidity decreases. Thus the total of treatment and drug savings ($537.7 million) exceeds treatment costs by $185.0 million (Table 4).

**Table 4:** National Program Resource Costs and Savings:

Training Staff	**$29,679,980**
Training Hypertensive cases	**$70,037,048**
Maintenance Visits	**$66,797,601**
Extra Drug Costs	**$186,182,588**
**TOTAL PROGRAM COSTS**	**$352,697,217**
Reduced Treatment	**$444,382,484**
Reduced drug Use	**$93,270,706**
**TOTAL SAVINGS**	**$537,653,190**
**NET PROGRAM SAVINGS**	**$184,955,973**

### Sensitivity analyses

Sensitivity analyses are reported on the parameters that were thought ex-ante to have the most potential to influence the costs and utilities.

Using no discount rate, decreasing the attainable risk reduction for each disease by 30%, or decreasing the percentage of persons successfully treated from 50% to 40%, all resulted in the project still being cost-saving. Even if the overall attained decrease in hypertension were to fall by half to 12.5%, the project would still be cost saving (saving $29 million) in addition to saving 28,052 DALYs. The cost-effectiveness indicators were particularly sensitive to the number of years people received dialysis. If the average number of years were to drop from 5 to 4 years then total DALYs would be 32,212 and cost savings would fall to $123 million. If time on dialysis would increase to six years, then DALYs saved would rise to 32,565 and cost-savings rise to $247 million.

## Discussion

Coronary heart disease and Cerebrovascular Accident (CVA) prevention are priority areas for improving health, especially in developed nations. World-wide targets have been set for the reducing the major Cardiovascular (CV) risk factors: Blood Pressure (BP), diabetes, dyslipidemia, smoking and diet in relation to obesity [28,29]. Nationwide projects are called for in order to significantly shift any nation's risk factor profile towards proven goals. Health policy and economic decisions makers in each country will be more likely to accept such large preventive projects not only if it the project is proven to save lifes and reduce morbidity, but also if it cost-effective (ie: having a low or even negative cost-per DALYs).

Our calculations show that implementation of a nationwide program to reduce hypertension, is likely to save over two thousand lives in the next 20 years, improve the quality of life in many more people and at the same time actually save $185.0 million in health care resources alone. The study is limited in the sense that it is using current estimates of relative risk and demographics to project disease incidence many years into the future. Economic changes over the next two decades are likely to influence the cost-utility ratio in so far as they will affect lifestyles (eg: may be increased obesity) which in turn will increase or decrease incidence of the diseases in question. In addition biases exist which tend to underestimate DALYs benefits as follows:-

a) Because mortality was only attributable for AMI and UAP for a one year post-hospitalization period. Deaths after this timeframe were conservatively not attributed to coronary syndromes. Similarly deaths due to stroke were very conservatively estimated because stroke deaths were only attributed to in-hospital mortality.

b) We based life years gained on the age specific life expectancies, unadjusted for a decrease of the disease in question. For example, if the program eradicates x% of AMIs, then life years saved should be based on a life expectancies adjusted upwards because of the eradication of x% of deaths from AMIs.

c) our model based its duration of ESRD solely on data from patients receiving dialysis. It is likely that this underestimates the true amount of life years saved as it excludes data from patients who had undergone transplants and had not reverted to dialysis [20]. DALYs benefits will be over or underestimated to the extent that fewer or more coronary events occur in February and March, the months on which the incidence survey was based [14]. The question whether there is a need for such a nationwide program is clear form many studies [2-5] which prove the need for risk factor control in order to achieve CV disease prevention. Despite this, there is growing evidence that the guidelines are not implemented enough and there is under treatment [5-7], poor patient compliance, physician's unawareness of the new recommendations [19], and non adherence to updating treatment and to targets of treatments [20,21].

Economic-Epidemiologic analysis has become a standard method for informing health policy makers, and is also helpful for individual physicians making medical decisions [28]. For each of the various CV risk factors, especially hypertension on account of the high prevalence, and the need for long-term, perhaps lifelong, treatment, quite minor changes in antihypertensive practices can have a significant impact on healthcare budgets [29].

Our first previous study, the Ashkelon Hypertension Detection and Control Program (AHDC Program), showed that a community approach to reduce cardiovascular mortality [9], during a 10-year period (1980–1990), can affect reduce CV morbidity and mortality. During an average of 6.4 years, follow-up BP, weight reduction, and smoking cessation, the standardized mortality ratio SMRs for MI, CVD and hypertensive diseases fell significantly. The project saved many life-years at no additional net cost to society. Also in our second project: the Israel Blood Pressure Control (IBPC) program [10], there was a significant increase in the percentage of patents defined as "controlled" in hypertension, Low Density Lipoprotein (LDL) levels and in the percentage of patients with diabetes.

It is important to note the impact of decreased mortality by estimating the additional Disability Adjusted Years of Life (DALYs) and not only life years added.

Each hypertension related disease encounters many measurable obstacles which patients face during their rehabilitation, which in many cases are not completely resolved. These diseases can cause pain, suffering, increasing dysfunction, temporary or permanent unemployment accompanied by attendant life stresses and major reductions in self image and in family status. In Israel, AMI patients are automatically absent from work for three months of rehabilitation as the minimum even if there are no AMI complications. This example shows that on top of the treatment and secondary prevention cost of each AMI there is also a considerable socioeconomic burden that may influence the total balance of the cost utility analysis.

Although probably underestimated the program is expected to result in 8,749 fewer AMIs, 8,134 fewer PVDs, 8,074 fewer CVAs, 6,765 fewer UAPs and 2,316 fewer cases of ESRD (Table 2). Around half of the estimated total of 2,242 fewer deaths will be due to the reduction in cerebrovascular strokes (1,114 fewer deaths), while there will be 543 fewer deaths from AMIs, 440 fewer deaths from ESRD and 145 fewer deaths from UAP. These translates into a total of 35,117 life years saved.

For CVA the whole socioeconomic benefits are even more likely to have been underestimated, as the program may also prevent work disability. The 1.7 relative risk of strokes in hypertensive patients [19] translates into a potential 41.2% reduction of strokes and stroke sequlae in successfully treated hypertensive cases. A further source of underestimation of DALYs saved is that our model based its duration of ESRD solely on data from patients receiving dialysis. It is likely that this underestimates the true amount of life years saved as it excludes data from patients who had undergone transplants and had not reverted to dialysis [20].

We feel that, consideration should be given to extending such a potentially cost-effective project (which saves life years, morbidity and resources) to a nationwide basis, in order to encompass as many people as possible.

## Conclusion

Our study shows that a national health education program to reduce cardiovascular risk factors, over the next 20 years, should succeed in decreasing the risk in one-half of AMI and UAP by 16.0%, CVA by 41.2%, CRF by 50.0% and PVD by 42.6%. The implementation of such a program nationwide is likely to save over two thousand lives, improve the quality of life in many more people and at the same time actually save $185.0 million in health care resources alone. It is conceivable that it may be extended not only throughout our country, but also to neighboring countries.

## Abbreviations

AHDC Ashkelon Hypertension Detection and Control

AMI Acute Myocardial Infarction

BP Blood Pressure

CV Cardiovascular

CVA Cerebrovascular Accident

DALYs Disability Adjusted Life Years

ESRD End stage renal disease

GDP Gross domestic product

HSV Health Status Valuation

IBPC Israel Blood Pressure Control

MOH Ministry of Health

LDL Low Density Lipoprotein

PVD Peripheral vascular disease

UAP Unstable Angina Pectoris

## Competing interests

The author(s) declare that they have no competing interests.

## Authors' contributions

CY instigated the project, designed, researched and wrote the introduction and discussion sections of the manuscript. GG designed, researched, built the model and wrote the methods and results sections of the manuscript.

RV instigated, designed and researched the project and commented on drafts of the paper. DD instigated, designed and researched the project and commented on drafts of the paper. DG instigated, designed and researched the project and commented on drafts of the paper.

## Appendix

See Tables: 5, 6 and 7.

**Table 5:** 

**Appendix I: Key Economic Variables**		**Reference/Data Source**
**ECONOMIC**		
Annual Discount Rate	3%	27
Exchange Rate: Shekels to USD (2005)	4.35	12
Per capita GDP (USD 2003)	$16,497	12
		
**Training Staff**		
Number of Clinics in Israel	3000	MOH Statistical Dept.
Staff per clinic receiving training	2	Ashkelon Project
Full Days Training in initial year	2	Ashkelon Project
Days training in subsequent years	1	Ashkelon Project
Cost per ticipant-day of training course	$100	Ashkelon Project
Tele-management & Operational costs per patient.	$60	Ashkelon Project
Nurse/GP guidance per patient in first year of program	$230	Ashkelon Project
		
**Unit Care Costs**		
Ambulatory visit to Nurse or Nutritionist	$7.83	15 mins visit & 5 mins secretarial time
Ambulatory GP visit	$11.37	15 mins GP & 5 mins secretarial time
Cost of OPV	$99.14	25% of general hospital cost (30)
General Hospital Cost per day	$397	30
ICU cost per day	$1,586	estimated as four times general per diem cost (30)
Rehabilitation Hospital Cost per day	$238.85	30
Home Physiotherapy Visit	$38.53	30
		
**Devices and Procedure costs**		
Stent	$202	Barzelai Hospital Accounts Dept.
Cypher	$2,989	Barzelai Hospital Accounts Dept.
Diagnostic Catheterisation	$2,464	30
Restenosis	$9,701	30, includes 1 day in ICU & 3 days in cardiac ward
Average cost of Catheterisation with Stent or cypher	$6,925	30
CABG operation	$12,740	30
TPA	$1,800	30 estimated as four times general per diem cost
PRS Streptokinaise	$81	31
Kidney Transplant Cost	$39,132	30
Haemodialysis cost	$313	30
Peritonea dialysis cost	$136	30
% receiving haemodialysis	88.8%	20
Average annual Dialysis Cost	$46,041	30
		
**Drug Costs**		
Post-MI or stroke drugs per day per patient- Males	$1.92	14
Post-MI or stroke drugs per day per patient- Females	$1.79	14
Post-UAP drugs per day per patient- Males	$1.92	14
Post-UAP drugs per day per patient- Females	$1.79	14
Correct preventive medication per day	$0.92	14, Ace inhibitor, Aspirin & Statin
Inadequate preventive medication per day	$0.31	14, Assumed only 1 correct drug taken

**Table 6:** 

**Appendix II: Key Epidemiologic Variables**		**Reference/Data Source**
**EPIDEMIOLOGIC**		
Male life expectancy at age 60 (years)	20.9	12
Female life expectancy at age 60 (years)	23.4	12
Healthy Adjusted Life expectancy at age 60 males	16.8	24
Healthy Adjusted Life expectancy at age 60 females	18.2	24
		
**Hypertension**		
% hypertensive cases, Males 25–44	3.8%	12
% hypertensive cases, Males 45–54	14.7%	12
% hypertensive cases, Males 55–64	27.8%	12
% hypertensive cases, Females 25–44	3.2%	12
% hypertensive cases, Females 45–54	13.9%	12
% hypertensive cases, Females 55–64	34.5%	12
% of persons treated successfully in program	50%	9,10,13
		
**Acute MI**		
Annual Incidence	8940	14
% AMI in Males	77.0%	14
20 year MI risk at age 25	5.0%	11
20 year MI risk at age 30	7.5%	11
20 year MI risk at age 35	10.0%	11
20 year MI risk at age 40	16.0%	11
20 year MI risk at age 45	22.0%	11
20 year MI risk at age 50	27.0%	11
20 year MI risk at age 55	32.0%	11
20 year MI risk at age 60	41.0%	11
20 year MI risk at age 65	50.0%	11
Annual increase in MI risk over 20 years	1.0%	Assumed
Decrease in risk of MI in persons successfully treated	16.0%	9,10,15–17
Health Status Valuation of MI patient	0.61	25
Years in decreased health state.	0.06	26
		
**Unstable Angina Pectoris**		
Annual Incidence	3354	14
% UAP in males	73.9%	14
Decrease in risk of UAP in persons succesfully treated	16.0%	9,10,15–17
Health Status Valuation of UAP patient	0.91	25
Years in decreased health state.	1.00	26
		
**Stroke**		
Mean age of stroke	73.2	19
% strokes in males	58.2%	19
Relative Risk of Stroke in Hypertensive cases	1.7	19
Stroke rate per 1000 aged 45–55	1.14	19
Stroke rate per 1000 aged 55–64	2.28	19
Stroke rate per 1000 aged 65–74	5.11	19
Stroke rate per 1000 aged 75–84	9.86	19
Decrease in Stroke risk in persons successfully treated	41.2%	19
Health Status Valuation of first ever stroke patient	0.78	25
Years in decreased health state.	7.80	26
		
**Chronic Renal Failure**		
ESRD incidence rates per 100,000 Males 45–54	31.9	19
ESRD incidence rates per 100,000 Males 55–64	77.8	19
ESRD incidence rates per 100,000 Males 65–74	148.7	19
ESRD incidence rates per 100,000 Males 75+	174.2	19
ESRD incidence rates per 100,000 Females 45–54	16.7	19
ESRD incidence rates per 100,000 Females 55–64	44.6	19
ESRD incidence rates per 100,000 Females 65–74	88.5	19
ESRD incidence rates per 100,000 Females 75+	71.9	19
Decrease in ESRD risk in persons successfully treated	49.9%	10
Health Status Valuation of ESRD	0.904	28
		
**PVD**		
PVD incidence per 1000 male hypertensive cases	6.20	23
PVD incidence per 1000 female hypertensive cases	3.65	23
PVD Relative Risk in Hypertensive to Normotensive cases	2.31	23
PVD Relative Risk in Hypertensive to controlled cases	1.40	23
Attainable risk reduction Hypertensive to Normotensive cases	56.8%	23
Attainable risk reduction Hypertensive to Controlled cases	28.4%	23
% treated who become controlled cases	50%	9,10
% treated who become normotensive cases	50%	9,10

**Table 7:** 

**Appendix III: Key Utilization variables**.			**Reference/Data Source**
**UTILIZATION**		Both Genders	
**Hypertension Treatment**			
% of persons transferred to NPC of hypertension		50%	9
Existing ambulatory visits in first year for hypertension		1.0	Barzelai Hospital
Existing annual maintenance visits for hypertension		0.5	Barzelai Hospital
Annual maintenance visits in program		2.0	9
Average time of visit (minutes)		15	9
	Males	Females	
			
**AMI Treatment**			
GP visits in three years prior to AMI	10	10	Assumption
% AMI that are catheterised	72%	59%	14
% AMI getting stent/baloon (primary PCI)	19%	14%	14
% AMI getting stent (late PCI)	38%	32%	14
% AMI getting CABG	8%	4%	14
% AMI being discharged after catheterisation	7%	9%	14
% AMI being discharged with no catheterisation	5%	22%	14
% AMI with Thrombolysis	23%	19%	14
% AMI rehospitalised for cardiac reasons in month	15.4%	15.7%	14
% ratio of cyphers to all cypher-stent usage	40.0%	40.0%	MOH Medical Technology Assesment Dept.

## References

[B1] Hajjar I, Kotchen TA (2003). Trends in prevalence, awareness, treatment, and control of hypertension in the United States, 1988–2000. JAMA.

[B2] Staessen JA, Gasowski J, Wang JG (2000). Risks of untreated and treated isolated systolic hypertension in the elderly: meta- analysis of outcome trials. Lancet.

[B3] Fedder DO, Koro CE, L'Italien GJ (2002). New National Cholesterol Education Program III guidelines for primary prevention lipid-lowering drug therapy: projected impact on the size, sex, and age distribution of the treatment-eligible population. Circulation.

[B4] (2002). American diabetes Association: clinical practice recommendation. Diabetes Care.

[B5] Pearson TA, Laurora I, Chu H, Kafonek S (2000). The lipid treatment assessment project (L-TAP): a multicenter survey to evaluate the percentages of dyslipidemic patients receiving lipid-lowering therapy and achieving low density lipoprotein cholesterol goals. Arch Intern Med.

[B6] EUROASPIRE (1997). A European Society of Cardiology survey of secondary prevention of coronary heart disease: principal results. EUROASPIRE Study Group. European Action on Secondary Prevention through Intervention to Reduce Events. Eur Heart J.

[B7] (2001). Clinical reality of coronary prevention guidelines : a comparison of EROASPIRE I and II in nine countries: EROASPIRE I and II Group. European Action on Secondary Prevention by Intervention to Reduce Events. Lancet.

[B8] Bernard DB, Townsend RR, Sylvestri MF (1998). Health and disease management: what is it and where is it going? What is the role of health and disease management in hypertension?. Am J Hypertens.

[B9] Yosefy C, Dicker D, Viskoper JR, Tulchinsky TH, Ginsberg GM, Leibovitz E, Gavish D (2003). The Ashkelon Hypertension Detection and Control Program (AHDC Program): a community approach to reducing cardiovascular mortality. Prev Med.

[B10] Yosefy C, Ginsberg GM, Dicker D, Viskoper JR, Tulchinsky TH, Leibovitz E, Gavish D, IBPC Investigators (2003). Risk factor profile and achievement of treatment goals among hypertensive patients from the Israeli Blood Pressure Control (IBPC) program – initial cost utility analysis. Blood Press.

[B11] Central Bureau of Statistics, Statistical Abstract of Israel 2006, No 57, Jerusalem. http://www1.cbs.gov.il/shnatonenew.htm.

[B12] Health Status in Israel 2003. Israel Center for Disease Control (ICDC) Pub. No. 235, 2004.

[B13] Fuchs Z, Viskoper JR, Drexler I, Nitzan H, Lubin F, Berlin S, Almagor M, Zulty L, Chetrit A, Mishal J (1993). Comprehensive individualised nonpharmacological treatment programme for hypertension in physician-nurse clinics: two year follow-up. J Hum Hypertens.

[B14] Acute Coronary Syndromes 2002, Israel Center for Disease Control (ICDC) publication Number 230 April 2003.

[B15] MacMahon S, Peto R, Cutler J, Collins R, Sorlie P, Neaton J, Abbott R, Godwin J, Dyer A, Stamler J (1990). Blood pressure, stroke, and coronary heart disease. Part 1, Prolonged differences in blood pressure: prospective observational studies corrected for the regression dilution bias. Lancet.

[B16] Wang JG, Staessen JA, Franklin SS, Fagard R, Gueyffier F (2005). Systolic and diastolic blood pressure lowering as determinants of cardiovascular outcome. Hypertension.

[B17] Andersen UO, Jensen G (2007). Decreasing population blood pressure is not mediated by changes in habitual physical activity. Results from 15 years of follow-up.

[B18] Women's Health in Israel, Israel Center for Disease Control (ICDC), 1999.

[B19] Bornstein NM, Aronovich BD, Karepov VG, Gur AY, Treves TA, Oved M, Korczyn AD (1996). The Tel Aviv Stroke Registry, 3600 Consecutive patients. Stroke.

[B20] (2004). Dialysis patients in Israel, follow-up indices 1989–2001. Israel Center for Dialysis and Kidney Transplants. Ministry of Health.

[B21] Chi-yuan Hsu, McCulloch CE, Durbinian J, Go AS, Iribarren C (2005). Elevated Blood Pressure and Risk of End-stage Renal Disease in Subjects Without Baseline Kidney Disease. Arch Intern Med.

[B22] Chobanian AV, Bakris GL, Black HR, Cushman WC, Green LA, Izzo JL, Jones DW, Materson BJ, Oparil S, Wright JT, Roccella EJ (2003). National Heart, Lung and Blood Institute Joint National Committee on Prevention, Detection, Evaluation and Treatment of High Blood Pressure. National High Blood Pressure Education Program Coordinating Committee: The Seventh Report of the Joint National Committee on Prevention, Detection, Evaluation and Treatment of High Blood Pressure; the JNC7 report. JAMA.

[B23] Kanel WB (1996). Blood Pressure as a cardiovascular risk factor. JAMA.

[B24] (2004). World Health Report. WHO, Geneva.

[B25] Murray CJL, Lopez AD, (eds) (1996). The Global Burden of Disease. WHO.

[B26] Murray CJL, Lopez AD (1996). Global Health Statistics. WHO.

[B27] (2001). WHO Commission on MacroEconomics and Health. Macroeconomics and health: investing in health for economic development. Report of the commission on Macroeconomics and health.

[B28] Fullard E, Fowler G, Gray M (1987). Promoting prevention in primary care: controlled trial of low technology, low cost approach. Br Med J (Clin Res Ed).

[B29] Berglund G, Eriksson KF, Israelsson B, Kjellstrom T, Lindgarde F, Mattiasson I, Nilsson JA, Stavenow L (1996). Cardiovascular risk groups and mortality in an urban swedish male population: the Malmo Preventive Project. J Intern Med.

[B30] (2005). Ministry of Health, Medical Services Price List. http://www.health.gov.il/english/Pages_E/default.asp?maincat=1&catId=8&PageId=8.

[B31] International Drug Price Indicator Guide. http://erc.msh.org/dmpguide/resultsdetail.cfm?.

